# Superficial Granulomatous Pyoderma Gangrenosum of the Penis: A Case Report

**DOI:** 10.1100/tsw.2006.376

**Published:** 2006-05-05

**Authors:** Shyamala S. Gopi, Alan T. Evans, Asif Raza, Derek J. Byrne

**Affiliations:** ^1^ Departments of Urology, Tayside University Hospitals, Ninewells Hospital, Dundee, UK; ^2^ Departments of Pathology, Tayside University Hospitals, Ninewells Hospital, Dundee, UK

**Keywords:** penis, superficial granulomatous variant of pyoderma gangrenosum, skin ulcer/pathology, topical immunosuppressant

## Abstract

Classic type of pyoderma gangrenosum (PG) is an uncommon ulceronecrotic cutaneous disease of uncertain aetiology characterised by broad zones of confluent ulceration with violaceous undermined margins. Some 50% of cases are associated with systemic diseases. The superficial granulomatous variant of pyoderma gangrenosum (SGPG) of the external genitalia is extremely rare Patients with this condition develop single or multiple ulcerated skin lesions often with sinus tract formation. The majority of these lesions were found on the trunk and limbs. SGPG is less likely to be associated with underlying disease processes than classic PG. We present a 58 year-old with recalcitrant penile ulceration demonstrated to be SGPG on biopsy. Although rare and poorly recognised, the histological features are sufficiently typical to allow the correct diagnosis to be established.

## INTRODUCTION

The classic type of pyoderma gangrenosum (PG) is an uncommon, ulceronecrotic, cutaneous disease of uncertain aetiology characterised by broad zones of confluent ulceration with violaceous, undermined margins. Some 50% of cases are associated with systemic diseases such as inflammatory bowel disease, vasculitis, Behcet's disease, sarcoidosis, rheumatoid and seronegative arthritis, myeloid leukaemia, and monoclonal gammopathy[1]. The lesion may develop at the site of trauma, a phenomenon known as pathergy[2]. The superficial granulomatous variant of pyoderma gangrenosum (SGPG) of the external genitalia is extremely rare as only one case of scrotal SGPG has been reported so far in the English literature[3] and none has been reported on the penis. Patients with this condition develop single or multiple ulcerated skin lesions often with sinus tract formation. Forty-five cases of SGPG or vegetative variant of PG have been sited and these lesions were sited on the face, forehead, breast, arms, back, abdomen, trunk, groin, or limbs. The majority of these lesions were found on the trunk and limbs[4]. SGPG is less likely to be associated with underlying disease processes than classic PG.

We present a 58-year-old male with recalcitrant penile ulceration demonstrated to be SGPG on biopsy. Although rare and poorly recognised, the histological features are sufficiently typical to allow the correct diagnosis to be established.

## CASE REPORT AND PATHOLOGICAL FINDINGS

A 58-year-old patient presented with a periurethral abscess. Following drainage, an urethrocutaneous fistula developed resulting in some 5 cm of anterior urethral loss, distal to penoscrotal junction (Figs. 1 and 2). The fistula failed to heal following insertion of urethral and suprapubic catheters. Biopsies were taken to exclude malignancy and tuberculosis. A gracilis flap was then utilised to reconstruct the urethra, but the graft failed due to postoperative infection with both pseudomonas and MRSA. The wound failed to heal despite a 3-month course of intravenous ciprofloxacin and teicoplanin and was, therefore, rebiopsied. The biopsy showed cutaneous tissue with widespread marked inflammation, located primarily

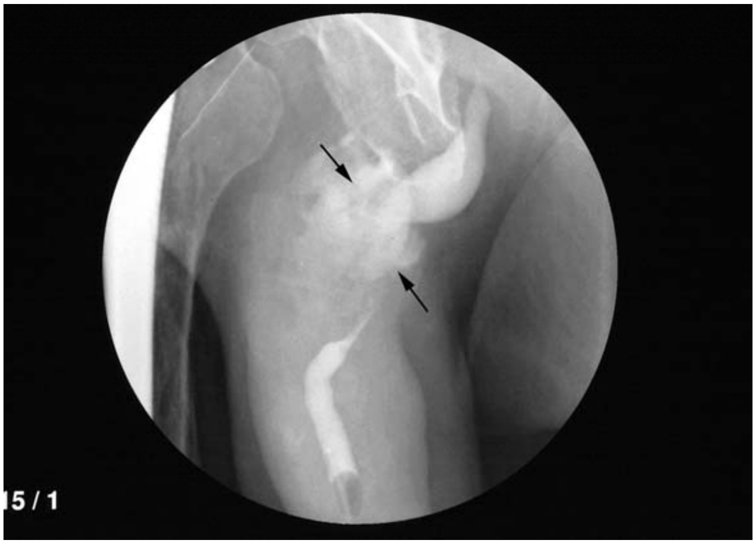

**FIGURE 1.** Cystogram: contrast fills a fairly short segment of urethra and then there is marked extravasation of contrast (arrows) through a sinus near the penoscrotal junction.

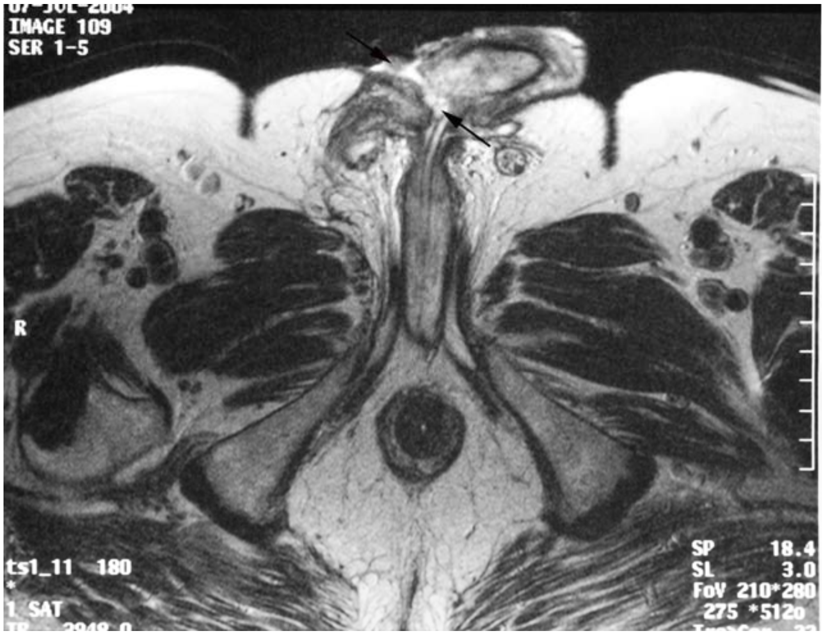

**FIGURE 2.** MRI pelvis with images centred on the perineum. A skin sinus track (arrows) is evident on the right side of the base of the penis. There was no deep-seated collection seen.

within the dermis, but mainly centred on sinus tracts which were lined by squamous epithelium. Tuberculosis and malignancy were excluded (Fig. 3).

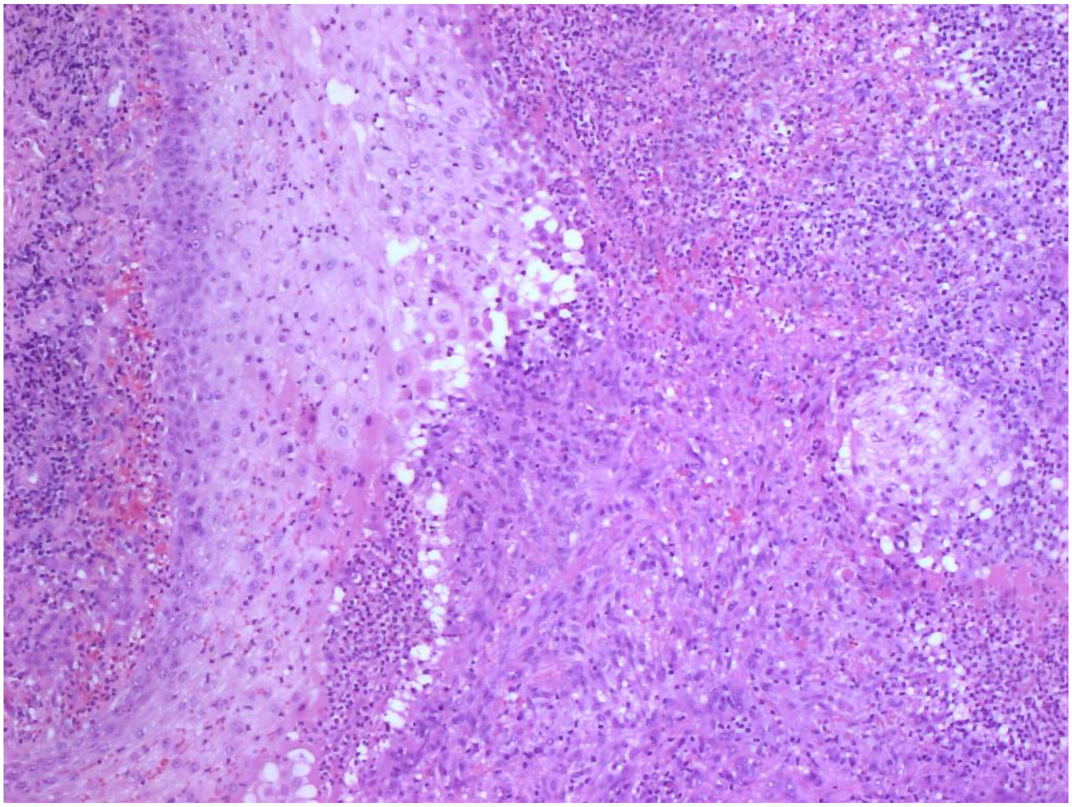

**FIGURE 3**. Combination of sinuses, abscesses, and a prominent plasma cell component combined with granulomatous inflammation suggests a diagnosis of SGPG.

Foci of abscess formation were apparent in close relation to the sinuses, but between the more acutely inflammed areas, chronic inflammatory cells predominated with aggregates of lymphocytes and plasma cells[5]. Poorly formed granulomata were also identified together with scattered multinucleated giant cells. There was no evidence of polarisable foreign debris. This spectrum of features was compatible with a diagnosis of SGPG. Any known associated diseases were excluded. The dermatologists instituted a course of topical steroids combined with the topical immunosupressant Tacrolimus. After 2 months, the wound has almost completely healed (Fig. 4).

## DISCUSSION

SGPG is a rare and severe inflammatory dermatosis occasionally presenting at genital sites. A rarity, even in dermatology practice, it is therefore likely to be overlooked or misinterpreted by urologist and histopathologists. The presence of squamous-lined sinus tracts with suppurative inflammation and sterile abscesses is similar to changes observed in hidradentitis suppurativa. These changes may well be reported as nonspecific. Bacterial, fungal, and mycobacterial infection should be excluded. The combination of sinuses, abscesses, and a prominent plasma cell component combined with granulomatous inflammation suggests a diagnosis of SGPG. Treatment of classic PG is immunosuppression with steroids +/– azothioprine or cyclosporine[6].

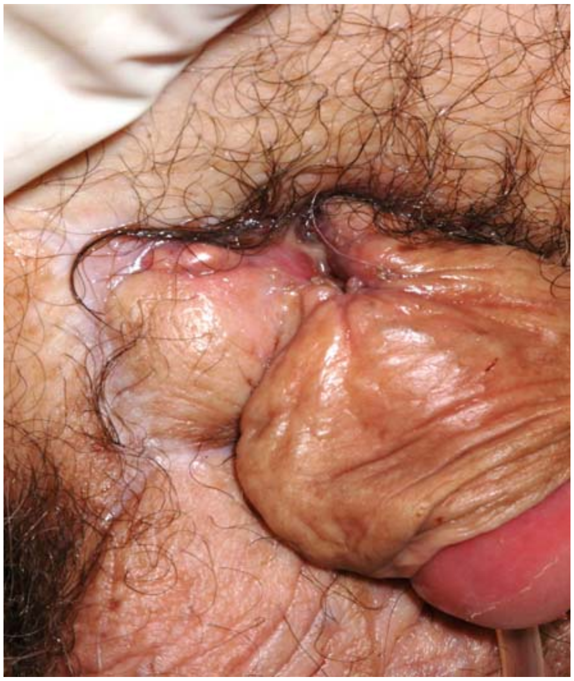

**FIGURE 4.** Healing SGPG of penis, 2 months after treatment with topical steroids and tacrolimus.

This case showed a satisfactory response to topical steroids and tacrolimus. Therefore, a high index of suspicion is necessary when a wound fails to heal satisfactorily. A multidisciplinary approach, involving the dermatologist, pathologist, and urologist is essential in the diagnosis and management of this rare and challenging condition. However, once recognised, standard treatment as described earlier allows satisfactory wound healing.
